# ARVO 2011: Visionary Genomics

**Published:** 2011-07

**Authors:** Hossein Nazari

**Affiliations:** Doheny Eye Institute, University of Southern California, Los Angeles, California, USA, Ophthalmology Department, Tehran University of Medical Sciences, Tehran, Iran

Fort Lauderdale, Florida once again hosted more than 10,000 ophthalmology and vision science researchers. The Association for Research in Vision and Ophthalmology (ARVO) annual meeting is well known for presentation of the latest milestones in fields related to vision science. ARVO is famous for being basic science oriented; clinical posters and papers, and even commercial exhibitions are markedly connected to molecular, immunologic and genetic aspects of disease and treatment modalities. Every year, ARVO brings established investigators and junior trainees from all over the world together and creates a unique environment to promote the next generation of ideas and idea makers. You can meet all the big names and researchers of tremendous insight at ARVO. For me, as a first-time attendee in such a science-oriented event, meeting colleagues and seeing such a wide range of state-of-the-art research was an invaluable experience.

ARVO is unique among other ophthalmology conferences for the non-formal attitude of the presenters and attendees. Even though the sessions are highly scientific in content and followed by challenging questions and answers, the casual attire of all attendees is eye catching at the very first look.

With the recent developments in molecular biology, genetics, and diagnostic imaging technologies, it is not surprising that ARVO has focused on these fields during the past decade. This year’s meeting was named “Visionary Genomics” with particular emphasis on molecular and clinical genetic aspects in the presentation, diagnosis, and management of ocular disease.

One of the differences of ARVO from similar meetings is the importance of poster sessions in this event. More than one thousand posters a day and a total of five thousand posters, all presenting original research, were presented this year. Posters were organized into correlated topics making a full and in-depth review of the latest advances in a specific field possible.

ARVO 2011 started on April 30, with a pre-congress day consisting of a couple of symposia and ophthalmic association meetings. The American Uveitis Society annual meeting was held one day before ARVO with presentations from all around the world. At the beginning of this meeting, Dr Narsing A Rao delivered a lecture titled “Photoreceptor mitochondrial oxidative stress and its prevention in uveitis.” He presented the latest progress regarding the rapidly evolving role of micro-RNAs in uveitis, a major part of which has been developed during the past several years by his research group. Micro-RNAs are small non-coding post-translational regulators of mRNA to which they attach and result in translational repression or silencing of the genes. By use of validated micro-RNA mimics, immunologic pathways have been manipulated to suppress the expression of cytokine genes, leading to a reduction in inflammation. It seems that micro-RNAs could be an important component of the next generation of therapeutic modalities.

The first day of ARVO 2011 was remarkable for the excellent presentation of “Innate and adaptive immunity in ocular defense and disease.” Our knowledge of the immune defense mechanisms in diverse conditions such as uveitis, allergy, transplantation rejection, tumor development, age-related macular degeneration (ARMD), and glaucoma has been improved immensely. We now know that these seemingly unrelated conditions are connected by common immunologic pathways and molecules. Overlapping mechanisms are creating a new generalized overview towards these diseases. The possibility of modifying common basic mechanisms along with availability of individual whole genome sequencing will hopefully lead to individualized preventive and therapeutic approaches.

Hot posters of the first day were too numerous to name, but included posters on nanobiodevices for drug delivery, artificial vision posters, individualized treatment modalities based on genetic testing, and new animal models for different ocular diseases.

I was able to attend some excellent oral presentations during the following days of ARVO 2011 and visit some posters and talk to their authors. Lectures and posters presenting results on ultrahigh resolution swept source- OCT (optical coherence tomography) imaging for measurement of retinal blood flow and oxygen tension, polarization sensitive-OCT, choroidal imaging with enhanced-depth OCT, VEGF (vascular endothelial growth factor) trap use for ARMD, real-time measurement of retinal temperature changes for calibration of laser power, complement factor H (CFH) polymorphism in primary open angle glaucoma (similar to what has previously been revealed for the CFH gene in ARMD), and results of various ARMD clinical trials were some of the interesting topics for me. Full access to the ARVO 2011 abstracts is possible through the official ARVO website (http://www.arvo.org).

Ocular inflammation and uveitis related studies were also exciting and mainly oriented towards the recognition of cellular components and molecular mediators of inflammation and possible mechanisms of their modulation for management of uveitis, the role of genetic factors (in particular micro-RNAs) in ocular inflammation, and results of various imaging techniques in uveitis syndromes. Long term results of immunomodulatory agents, including commercially available anti-TNF (anti-tumor necrosis factor) medications for management of intraocular inflammation, were also presented in the meeting.

On the last day of the meeting and in one of the main uveitis sessions, we presented our results on prognostic variables for uveitic epiretinal membranes (ERMs) with a morphometric spectral domain OCT approach; we showed that uveitic ERM thickness increases with time, even in clinically inactive eyes. These changes probably indicate ongoing subclinical inflammation which requires more aggressive treatment modalities to prevent long term complications.

Even though American or European-based Iranian ophthalmologists and basic science researchers had a significant contribution to the conference, very few Iranian colleagues attended this year’s ARVO. This limits our clinician-researcher society and eventually our patients from taking full advantage of this excellent scientific meeting. Next year’s ARVO meeting will be held on May 6–10, 2012 in Fort Lauderdale, Florida and we look forward to more presentations from Iran.

